# High Entropy Spinel Oxide (AlCrCoNiFe_2_)O as Highly Active Oxygen Evolution Reaction Catalysts

**DOI:** 10.1021/acsomega.4c03807

**Published:** 2024-06-13

**Authors:** Pouria Dadvari, Wei-Hsuan Hung, Kuan-Wen Wang

**Affiliations:** Institute of Materials Science and Engineering, National Central University, No. 300 Jhong-da Rd., Jhongli City, Taoyuan County 320, Taiwan

## Abstract

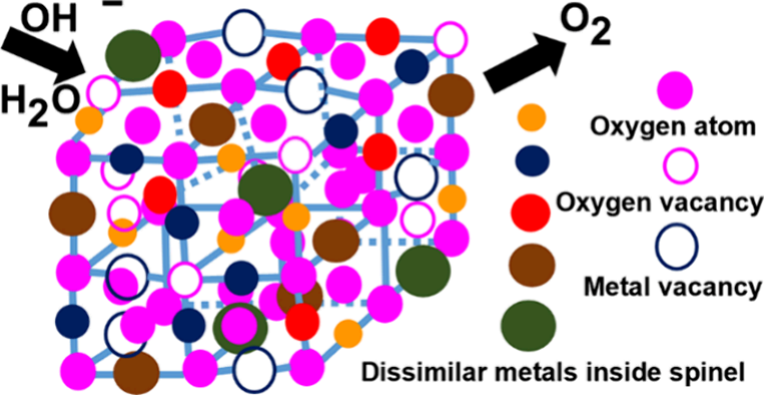

The advancement of water electrolyzer technologies and the production of sustainable hydrogen fuel heavily rely on the development of efficient and cost-effective electrocatalysts for the oxygen evolution reaction (OER). High entropy ceramics, characterized by their unique properties, such as lattice distortion and high configurational entropy, hold significant promise for catalytic applications. In this study, we utilized the sol–gel autocombustion method to synthesize high entropy ceramics containing a combination of 3d transition metals and aluminum ((AlCrCoNiFe_2_)O). We then compared their electrocatalytic performance with other series of synthesized multimetal and monometallic oxides for the OER under alkaline conditions. Our electrochemical analysis revealed that the high entropy ceramics exhibited excellent performance and the lowest charge transfer resistance, Tafel slope (29 mV·dec^–1^), and overpotential (η_10_ = 230 mV). These remarkable results can be primarily attributed to the high entropy effect induced by the addition of Al, Cr, Co, Ni, and Fe, which introduces increased disorder and complexity into the material’s structure. This, in turn, facilitates more efficient OER catalysis by providing diverse active sites and promoting optimal electronic configurations for the reaction. Furthermore, the strong electronic interactions among the constituent elements in the metallic spinels further enhance their catalytic activity in the initiation of the OER process. Combined with the reduced charge transfer resistance, these factors collectively play pivotal roles in enhancing the OER performance of the electrocatalysts. Overall, our study provides valuable insights into the design and development of high-performance electrocatalysts for sustainable energy applications. By harnessing the high entropy effect and leveraging strong electronic interactions, electrocatalytic materials can be tailored to improve efficiency and stability, thus advancing the progress of clean energy technologies.

## 1 Introduction

The production of hydrogen through electrochemical water splitting has emerged as a promising avenue for addressing the energy crisis and mitigating global warming.^1,2^ In this process, the anodic reaction of oxygen evolution is pivotal as it is the rate-determining step during water splitting. Efficient catalysts are essential to promoting the kinetics of this oxygen evolution reaction (OER), thereby enhancing the overall efficiency of water splitting. However, developing low-cost, active, and stable electrocatalysts for OER presents several formidable challenges.^3,4^ One of the primary hurdles is overcoming the high activation energy required for the reaction to proceed. This activation energy barrier impedes the rate of the OER, which limits the efficiency of hydrogen production. Additionally, facilitating electron transfer between the electrolyte and the catalyst surface is crucial for promoting the OER kinetics.^5^ Without efficient electron transfer, the catalyst’s activity is severely hampered.

The effectiveness of OER catalysts is influenced by various factors including their composition, structure, size, morphology, and conductivity.^5^ Optimizing these parameters is vital for achieving a high catalytic activity. Researchers have explored a diverse range of catalysts comprising non-noble, earth-abundant metals as alternatives to expensive noble metal oxides like IrO_2_ and RuO_2_.^6−9^ Some of these alternative catalysts have demonstrated superior activity, particularly in alkaline environments, offering potential solutions for cost-effective hydrogen production.^10^ Incorporating multiple metals into the catalyst’s oxide structure represents a promising strategy for enhancing catalytic performance. This incorporation can induce changes in the catalyst’s electronic configuration through strain or ligand effects.^11,12^ These modifications influence the overlap between atomic orbitals and the electrostatic force environment, thereby affecting the catalyst’s activity.^13,14^ Furthermore, the introduction of foreign ions with dissimilar valences and sizes into the crystallographic structure can create defects and charge vacancies. These structural alterations can significantly impact the catalytic properties of the electrocatalyst.^15^

High entropy ceramics represent a fascinating class of materials garnering significant attention, particularly for catalytic applications.^16,17^ These ceramics are characterized by their high configurational entropy, which imparts structural stability, lattice distortion, defective structure, and a cocktail effect arising from the combination of multiple elements. This unique combination of properties makes them promising candidates for various catalytic processes.

Among high entropy ceramics, spinel compounds stand out due to their robust electrical conductivity and stability, particularly in alkaline solutions.^18^ Yang et al. have synthesized numerous catalysts by incorporating additional metals into the spinel structure, and integrating second, third, and even fourth metals into the spinel structure has been explored to promote OER activity.^19^ This approach capitalizes on the synergistic effects between different metal elements, leveraging their unique properties to improve catalytic performance.^20^ By carefully selecting the combination of metals and controlling their incorporation into the spinel lattice, researchers can tailor the catalyst’s properties to optimize OER activity and stability.^6,10,21−23^

In this study, we employed an autocombustion method^24^ to synthesize a series of multimetal and monometallic oxides, aiming to develop effective electrocatalysts for the OER. This method offers distinct advantages, including simplicity, scalability, and precise control over the composition and morphology of the resulting materials. Through systematic exploration of the structural properties of these oxides, we made a significant finding. Among the synthesized materials, (AlCrCoNiFe_2_)O exhibited a highly promising performance in alkaline media. Specifically, it demonstrated an overpotential of 230 mV at 10 mA·cm^-2^ and a Tafel slope of 29 mV·dec^-1^. These results underscore the remarkable catalytic activity of (AlCrCoNiFe_2_)O in promoting the OER. In addition, this observation highlights the synergistic effects of various factors contributing to the superior performance of (AlCrCoNiFe_2_)O. The high entropy effect, strong electronic interactions among the constituent elements, and reduced charge transfer resistance collectively play pivotal roles in enhancing the OER performance. Overall, our study provides valuable insights into the design and development of high-performance electrocatalysts for sustainable energy applications.

## 2 Experimental Section

### 2.1 Preparation of the Metal Oxide Catalysts

Sol–gel autocombustion routes were used to prepare metal oxide catalysts. Stoichiometric amounts of Al(NO_3_)_3_·H_2_O, Ni(NO_3_)_2_·6H_2_O (Alfa Aesar), Cr(NO_3_)_3_·9H_2_O, Fe(NO_3_)_3_·9H_2_O (Sigma-Aldrich), and Co(NO_3_)_2_·6H_2_O (SHOWA) were weighed and dissolved inside DI water. Citric acid C_3_H_4_(OH)(COOH)_3_ (SHOWA) was used as the fuel with a fuel:total metal ion ratio of 1.5:1. For the preparation of (AlCrCoNiFe_2_)O, (AlCoNiFe_2_)O, (CoNiFe_2_)O, and CoFe_2_O_4_, metal ratios were Al:Cr:Co:Ni:Fe; 1:1:1:1:2, Al:Co:Ni:Fe; 1:1:1:2, Co:Ni:Fe; 1:1:2, and Co:Fe; 1:2, as shown in Table S1 in the Supporting Information. The pH value of the solutions was adjusted to around 7 by the addition of ammonium hydroxide (NH_4_OH). Solutions were put inside an oven at 130 °C for 10 h to vaporize water and make the gel. Finally, the formed gels in glass beakers were placed on a hot plate at a temperature of 450 °C to make the combustion. After the combustion, ashes were collected and ground with a mortar and a pestle.

### 2.2 Characterizations of Metal Oxide Catalysts

Phase identification of samples was obtained by an X-ray diffractometer (D8-Bruker XRD) with λKα equal to 1.54 (Å) and copper anode operated at 40 kV and 40 mA. Surface chemical compositions of metal oxide catalysts were analyzed by X-ray photoelectron spectrometry (XPS, Thermo Fisher, Thermo VG-Scientific, Sigma Probe), with an Al Kα X-ray source at 1486.6 eV. The binding energies of XPS were referenced to a C 1s with a peak at 284.27 eV. To verify the microstructure, morphology, and elemental distribution of metal oxide catalysts, transmission electron microscopy (TEM, JEM-2000FXII, operated at 200 kV) and scanning electron microscopy (SEM, HITACHI SU-8200, operated at 20 kV) equipped with an energy-dispersive X-ray spectroscopy detector (EDS) were applied.

### 2.3 Electrochemical Measurement

All of the electrochemical measurements were performed using CHI705E (T1438 Inc. USA) with the nickel foam (NF) substrate for the working electrode and platinum wire and Hg/HgO as the counter and reference electrodes, respectively, at ambient temperature and pressure (25 °C and 1 atm, respectively) inside a 1 M KOH electrolyte. Each synthesized powder (10 mg) was dissolved in 500 μL of isopropanol and 10 μL of Nafion and then sonicated for 20 min. Nickel foams were cleaned by sonication first with acetone, then with ethanol each time for 30 min, and finally with DI water for 10 min. Mass loading of the catalysts on both sides of the porous nickel foam (100 μL of prepared inks was dropped on NF with a 2 cm^2^ geometric area) was equal to 0.98 mg·cm^–2^.

Another kind of binder (for the chronopotentiometry test) containing 20 mg of metal oxide powder dissolved in 1-methyl-2-pyrrolidinone (99% extra pure, ACROS) was also used. This mixture was combined with a binder (containing 70 mg of poly(vinyl alcohol) 98%, Alfa Aesar, dissolved in DI water, which was heated at 120 °C for 30 min) and sonicated for 30 min to make the ink. Mass loading equal to 0.4 mg·cm^–2^ with the NF substrate and platinum sheet (1.5 × 1.5 cm^2^ counter electrode) was used for 100 h of chronopotentiometry with a 10 mA constant current.

An accelerated durability test (ADT) with 1 V·s^–1^ for 1000 cycles and chronoamperometry at 1.77 V (vs RHE) was done for stability comparison of catalysts. Linear sweep voltammetry (LSV) curves were performed with a 5 mV·s^–1^ scan rate with 100% compensated solution resistance. Electrochemical impedance spectroscopy (EIS) was done in the frequency range from 0.1 to 10^5^ Hz with a 5 mV amplitude.

All potentials reported were converted to RHE by using the equation:
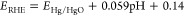
1

Overpotential was obtained by subtracting the thermodynamic potential of the OER from the real potential of experiments:
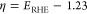
2

## 3 Results and Discussion

**Figure 1:**
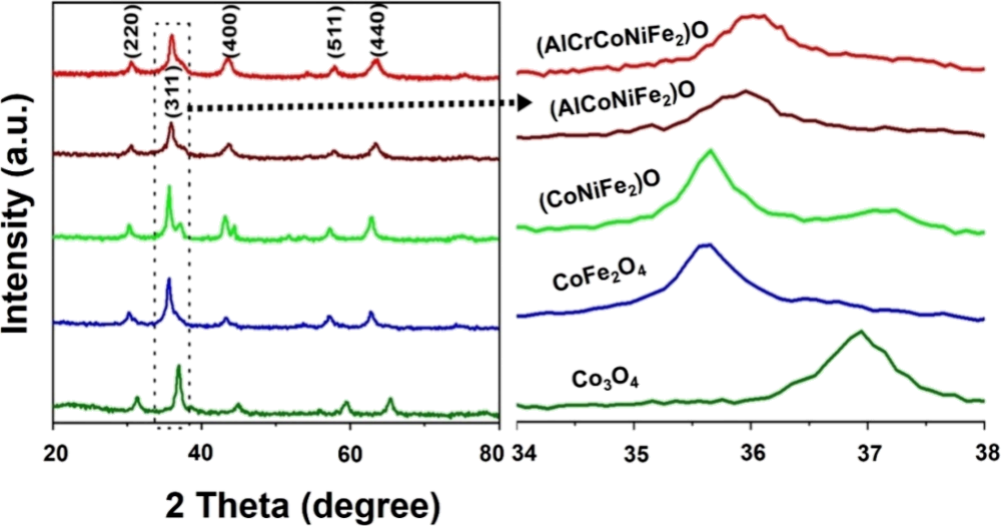
XRD pattern of metal oxide catalysts and the shifts in their main peak (311) by the incorporation of metals with dissimilar sizes.

The X-ray diffraction patterns of Co_3_O_4_, CoFe_2_O_4_, (CoNiFe_2_)O, (AlCoNiFe_2_)O, and (AlCrCoNiFe_2_)O are shown in Figure 1. Peaks around 2θ = 31, 36, 43.2, 58, and 63° are attributed to the (220), (311), (400), (511), and (440) planes, respectively, of the cubic spinel lattice. This crystallographic arrangement aligns with the “*Fd*3̅*m*” space group (JCPDS #750198), as referenced in Figures S1–S5 of the Supporting Information. A noteworthy observation is the shifting of these diffraction peaks toward lower angles in spinels such as CoFe_2_O_4_ and others [(CoNiFe_2_)O, (AlCoNiFe_2_)O, and (AlCrCoNiFe_2_)O] when compared to pure Co_3_O_4_. This phenomenon can be attributed to the incorporation of additional metal ions (Cr, Ni, and Fe) into the crystal structure, resulting in an expansion of the lattice parameters. These incorporated ions possess larger atomic sizes compared to Co, thereby influencing the overall lattice geometry and causing a systematic decrease in the diffraction angles. Moreover, the minor peak at approximately 37.2° corresponds to the (222) plane of the cubic spinel, a characteristic present across all spinel structures (more pronounced in Co_3_O_4_ and (CoNiFe_2_)O, as shown in Figures S3 and S5). However, the intensity of this peak diminishes with the incorporation of multiple metals in other spinels, indicating an anomalous reduction in XRD peak intensity due to lattice distortion induced by metal additions.^25^

**Figure 2:**
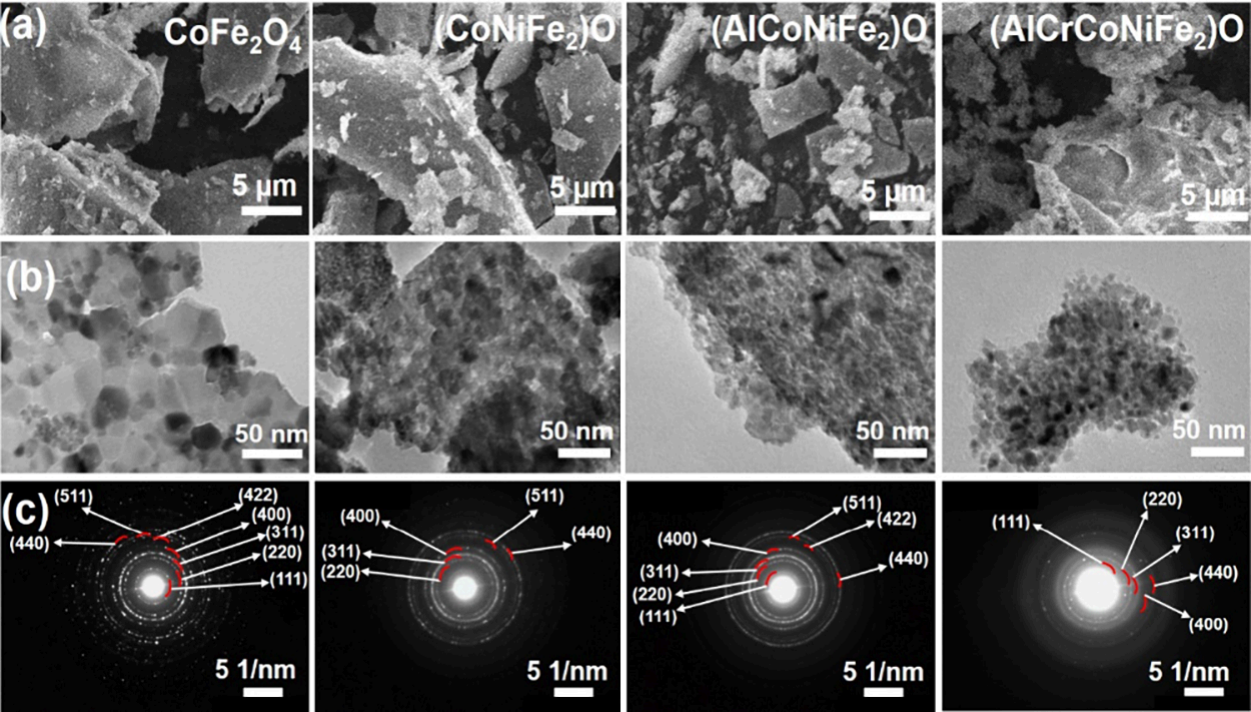
(a) SEM micrographs, (b) TEM micrographs, and (c) electron diffraction patterns of metal oxide catalysts.

The morphological characteristics of metal oxide catalysts are depicted in Figure 2; as the number of incorporated metals or the entropy of the system increases, there is a discernible trend toward smaller particle sizes across the spinel series. Particularly worth noting are the spinels (AlCrCoNiFe_2_)O and (AlCoNiFe_2_)O, which exhibit remarkably smaller particle sizes of less than 10 nm. This reduction in particle size can be attributed to the intricate interplay between the diverse metallic constituents within the spinel lattice, which introduces structural heterogeneity and ultimately influences the catalytic performance. Furthermore, the electron diffraction patterns obtained for all spinels indicate a polycrystalline nature for each metal oxide catalyst, highlighting the presence of multiple crystalline domains within the material. This polycrystalline structure is consistent with the expected arrangement in spinel-based materials, where crystallites with varying orientations contribute to the overall material composition. On the other hand, the energy-dispersive X-ray (EDS) elemental mapping depicted in Figure S6 illustrates a nearly uniform distribution of oxygen and metals throughout (AlCrCoNiFe_2_)O.

**Figure 3:**
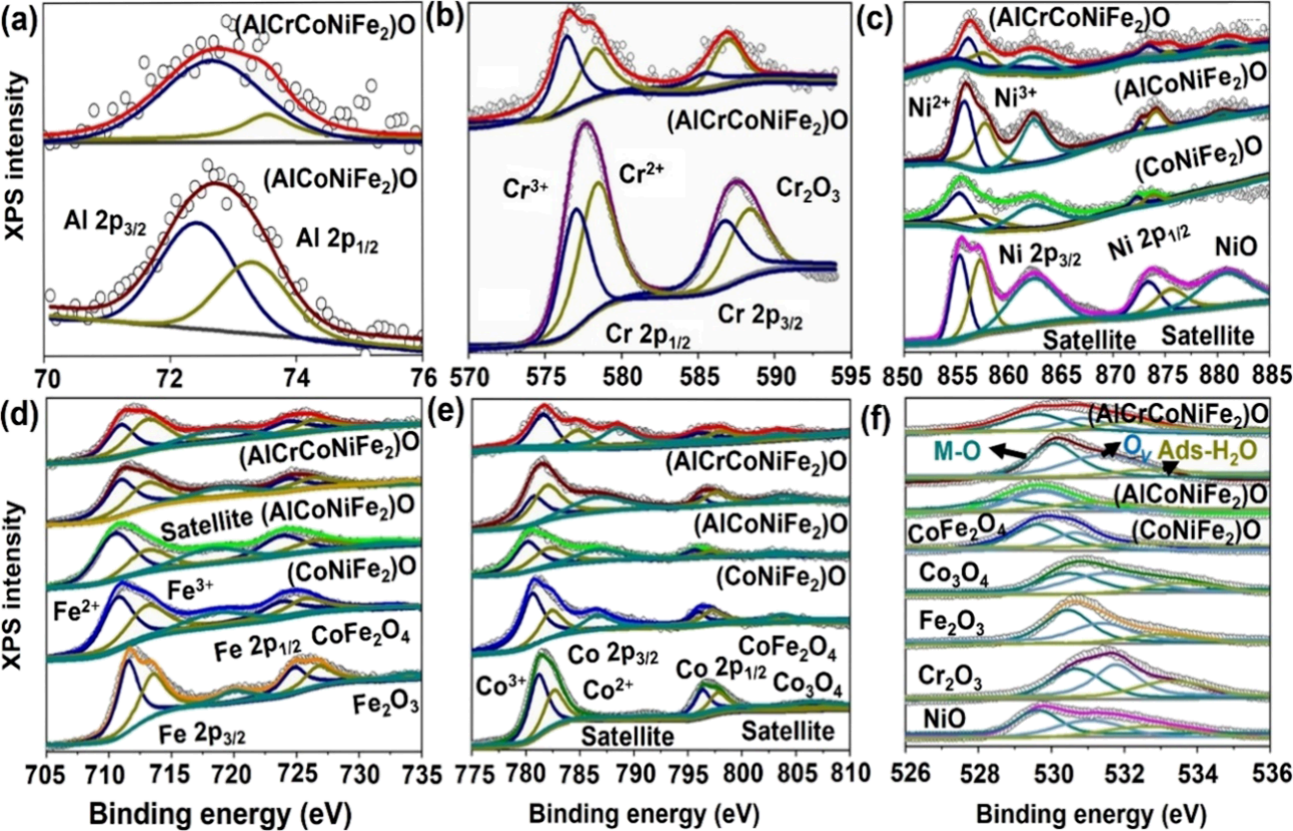
XPS spectra of metal oxide catalysts and reference samples at (a) Al 2p, (b) Cr 2p, (c) Ni 2p, (d) Fe 2p, (e) Co 2p, and (f) O 1s.

Additionally, the X-ray photoelectron spectroscopy (XPS) results presented in Figure 3 offer further insights into the chemical composition and electronic properties of the oxides under investigation. Analysis revealed the presence of Al, Cr, Fe, Co, and Ni as mixed oxides within the samples. Notably, in multimetal oxide catalysts, Co and Fe are found in higher oxidation states (+3) on the catalyst surface compared to the reference sample. This leads to a decrease in the total electron density within the outer shell of metals. Consequently, this reduction has the potential to alter the adsorption strength of oxygen intermediates such as MOH, MO, and MOOH, which are formed during the OER process, thereby enhancing the OER performance.^26^ Moreover, a comparative study against reference samples such as Cr_2_O_3_, Fe_2_O_3_, Co_3_O_4_, and NiO unveils notable peak shifts in the multimetal oxides, indicating intricate electron transfer phenomena among all components. These observed peak shifts are not confined to the metals alone but extend to the O 1s spectra, as well. Here, distinct peaks corresponding to M–O bonds, coordinated oxygen (or oxygen vacancies), and adsorbed H_2_O molecules of multimetal oxides deviate from the original locations at 528, 530, and 531.8 eV, respectively. This observation reinforces the notion of strong electronic interactions within the multimetal oxide catalysts. Combining the above findings from XRD, TEM, and XPS characterizations, it becomes evident that the multimetal oxide catalysts, particularly (AlCrCoNiFe_2_)O, possess a polycrystalline nature with multiple crystalline domains. Moreover, the pronounced electronic interactions among all constituents within the cubic spinel lattice underscore the complex and synergistic nature of these materials, which likely contributes to their enhanced catalytic performance.^27,28^

**Figure 4:**
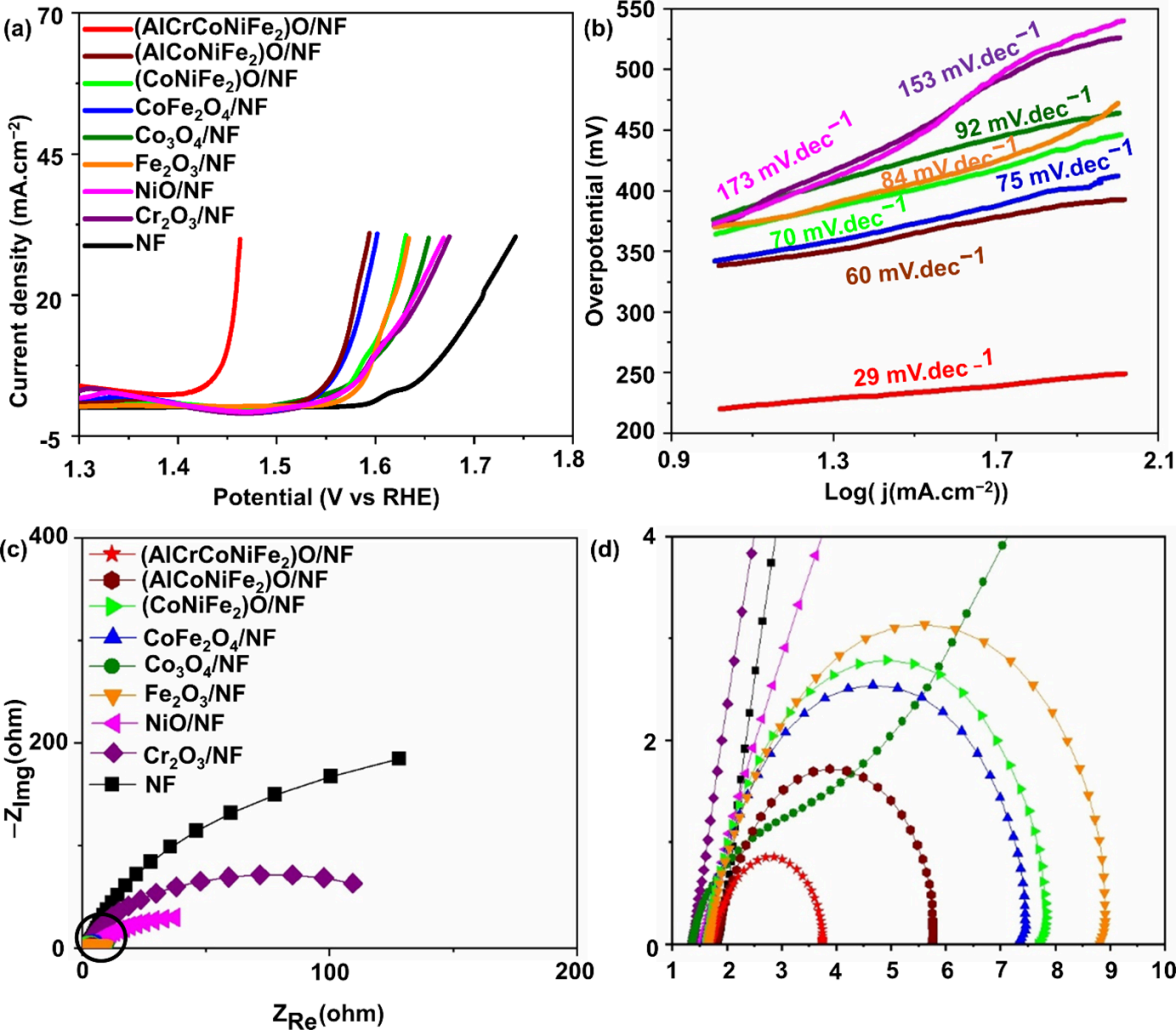
OER performance. (a) LSV curve, (b) Tafel slope, (c) Nyquist plot recorded at 1.566 V (vs RHE), and (d) magnified region in panel (c) for metal oxide catalysts and references in 1 M KOH.

Figure 4 illustrates the LSV curves of metal oxide catalysts and references during the OER in 1 M KOH. Notably, the obtained overpotentials at 10 mA·cm^–2^ (η_10_) reveal significant variations among the tested materials. For instance, (AlCrCoNiFe_2_)O exhibits the lowest overpotential at η_10_ = 230 mV followed by (AlCoNiFe_2_)O (338 mV), (CoFe_2_)O (342 mV), and (CoNiFe_2_)O (365 mV). In contrast, the reference monometallic oxides (Fe_2_O_3_, Co_3_O_4_, NiO, and Cr_2_O_3_) display higher overpotentials ranging from around 370 mV to larger values. This comparative analysis underscores the notable enhancement in catalytic activity achieved through the addition of multiple metals/oxides, leading to a substantial reduction in overpotentials. Of particular interest is the significant decrease in overpotential observed upon the addition of Cr into (AlCoNiFe_2_)O, resulting in a reduction of approximately 100 mV. This notable enhancement can be attributed to the high entropy effect induced by the addition of Cr, which introduces increased disorder and complexity into the material’s structure, thereby facilitating more efficient OER catalysis.^29,30^

Furthermore, the Tafel slopes extracted from the data corroborate the superior performance of (AlCrCoNiFe_2_)O, exhibiting the lowest value at 29 mV·dec^–1^.

This performance surpasses most multimetal oxide catalysts reported in the literature (see Table S2), underscoring the exceptional catalytic prowess of (AlCrCoNiFe_2_)O. Here, the presence of metals in octahedral or tetrahedral sites may serve as primary active sites, as dictated by the adsorbate evolution mechanism (AEM).^30^ Experimental electrochemical impedance spectroscopy (EIS) data, as illustrated in Figure 4c,d, further support these findings by indicating that (AlCrCoNiFe_2_)O possesses the lowest charge transfer resistance among the tested materials. This observation underscores the combined influence of the high entropy effect,^31−34^ strong electronic interactions, and reduced charge transfer resistance^35^ in promoting the superior OER performance of (AlCrCoNiFe_2_)O.

**Figure 5:**
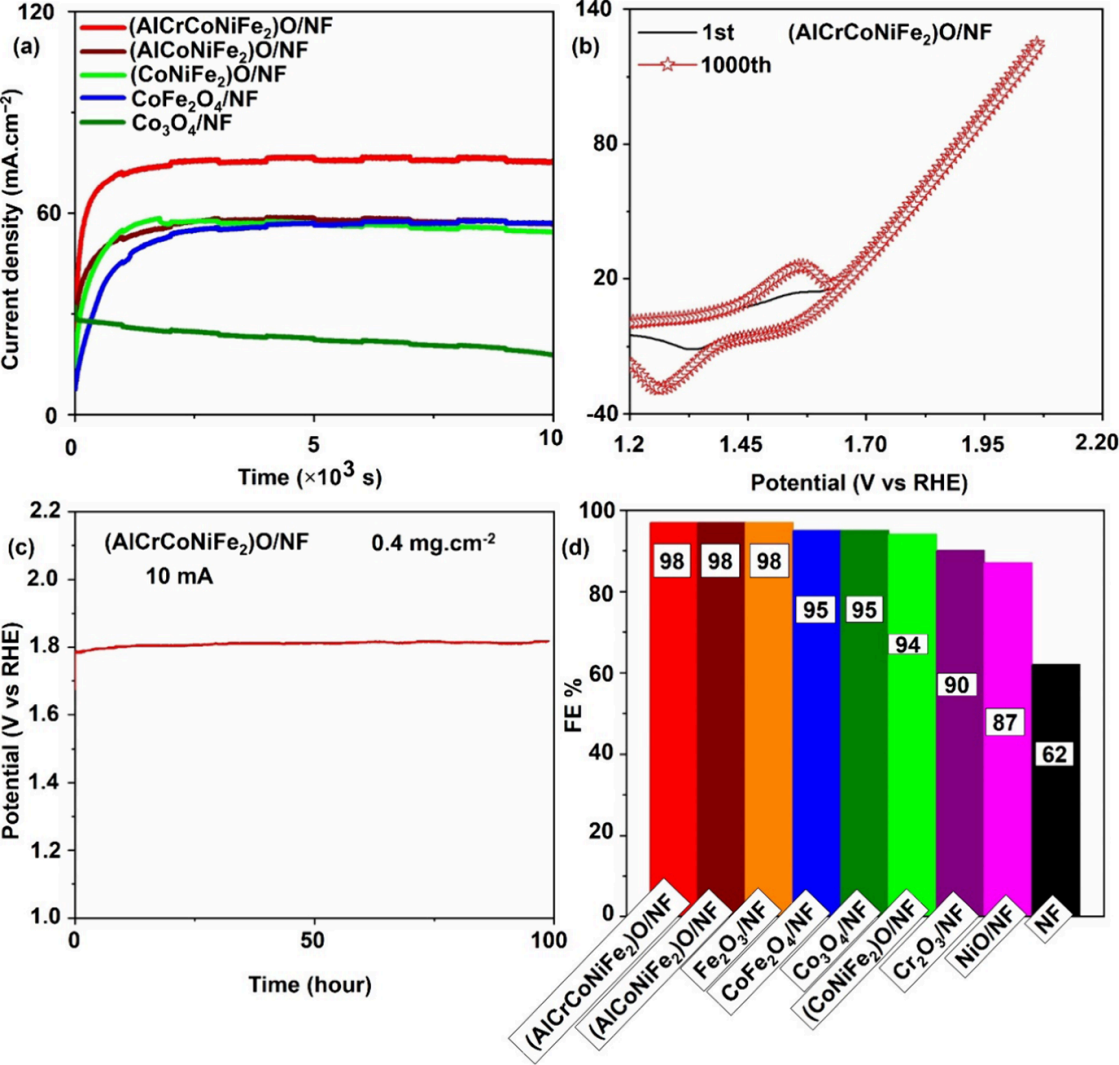
Stability and efficiency of catalysts for OER. (a) Chronoamperometry at 1.77 V (vs RHE), (b) ADT for 1000 cycles, (c) chronopotentiometry at 10 mA with uncompensated solution resistance, and (d) Faraday efficiency at 1.76 V (vs RHE) for 4000 s.

The chronoamperometry test conducted in the faradaic region, as depicted in Figure 5a, serves as a critical assessment of the stability of multimetal oxides for the OER in alkaline electrolytes. At a voltage of 1.77 V, (AlCrCoNiFe_2_)O emerges as the standout performer, exhibiting the highest current density among all of the tested materials. This observation suggests that (AlCrCoNiFe_2_)O possesses the lowest charge transfer resistance and demonstrates superior stability compared to other multimetal oxides under similar conditions. To further evaluate the long-term durability of (AlCrCoNiFe_2_)O, an accelerated durability test (ADT) with 1000 cycles was conducted, as illustrated in Figure 5b. The increase in the peak area of the redox cyclic voltammetry (CV) curves for (AlCrCoNiFe_2_)O can be attributed to the accumulation of charges and strengthening of the local electric field during successive redox cycles. Importantly, the overlapping of the 1st and 1000th CV curves demonstrates the robust durability of (AlCrCoNiFe_2_)O in an alkaline environment, indicating minimal degradation or loss of catalytic activity over extended cycling. Furthermore, the stability assessment over an extended duration reveals that (AlCrCoNiFe_2_)O maintains its performance with less than a 40 mV increase in overpotential over 100 h, as shown in Figure 5c. This remarkable stability underscores the suitability of (AlCrCoNiFe_2_)O for prolonged operation in OER applications, offering reliability and longevity in practical settings. Moreover, (AlCrCoNiFe_2_)O exhibits high efficiency for oxygen production, as supported by the Faraday efficiency measurements depicted in Figure 5d. This finding further validates the exceptional electrocatalytic performance of (AlCrCoNiFe_2_)O, affirming its potential as a promising candidate for scalable and efficient oxygen evolution processes.

## 4 Conclusions

In this study, by taking the advantages of high entropy ceramics, such as lattice distortion and high configurational entropy, the sol–gel autocombustion technique has been applied to fabricate high entropy ceramics comprising Al, Cr, Co, Ni, and Fe ((AlCrCoNiFe_2_)O). Subsequently, their OER performance against other sets of synthesized multimetal and monometallic oxides under alkaline conditions has been studied. Our electrochemical assessment unveiled the superior performance of the high entropy ceramics, exhibiting the lowest charge transfer resistance, Tafel slope (29 mV·dec^–1^), and overpotential (η_10_ = 230 mV). These outstanding outcomes can largely be ascribed to the high entropy effect, electronic interactions among the constituent elements in the ceramics, and low charge transfer resistance, which collectively assume pivotal roles in promoting the OER performance of the electrocatalysts. Overall, our study imparts invaluable insights into the design of high-performance electrocatalysts for sustainable energy applications. By harnessing the high entropy effect and capitalizing on strong electronic interactions, electrocatalytic materials can be tailored to enhance efficiency and stability, thus propelling the advancement of clean energy technologies.

## Supporting Information Available

Additional experimental methods and results including XRD, SEM, and TEM data (PDF)
