# Supplementary material for: High Entropy Spinel Oxide (AlCrCoNiFe2)O as Highly Active Oxygen Evolution Reaction Catalysts
Source: ACS Omega. 2024 Jun 13;9(25):27692–8. doi: 10.1021/acsomega.4c03807 (PMC11209678; doi:10.1021/acsomega.4c03807)
Supplement: Supplementary file 1 — ao4c03807_si_001.pdf [file ao4c03807_si_001.pdf]

Supplementary information

High Entropy Spinel Oxide $(AlCrCoNiFe_2)O$ as Highly Active Oxygen Evolution Reaction Catalysts

Pouria Dadvari, Wei-Hsuan Hung* and Kuan-Wen Wang*

Institute of Materials Science and Engineering, National Central University, No. 300 Jhong-da Rd., Jhongli City 320, Taoyuan County, Taiwan

Corresponding authors:

*Wei-Hsuan Hung, E-mail: hungwh@ncu.edu.tw

*Kuan-Wen Wang, E-mail: kuanwen.wang@gmail.com

Mixing or configurational entropy

The mixing entropy of oxides can be estimated using the following formula: [1]

$$\Delta S_{config} = -R\sum_{i=1}^{n} c_i \ln c_i \quad (S1)$$

All spinel can be considered to have the same total metal over oxygen (0.75) ratio like normal spinel AB_2O_4 (A and B are metals and O is oxygen).

Faraday efficiency measurement

Experimental amounts of oxygen were collected by water displacement and chronoamperometry for 4000 seconds at 1.76 V (vs. RHE). The theoretical quantity of oxygen produced was calculated from the same I-t curve using equations:

$$n_{O2-exp} = \frac{P_{O2}V_{O2}}{RT} \quad (S2)$$

$$P_{O_2} + P_{1M-KOH} = P_{air} = 1atm \quad (S3)$$

$$n_{O2-theo} = \frac{\int_0^t Idt}{n \times F} \quad (S4)$$

$$FE\% = \frac{n_{O2-exp}}{n_{O2-theo}} \quad (S5)$$

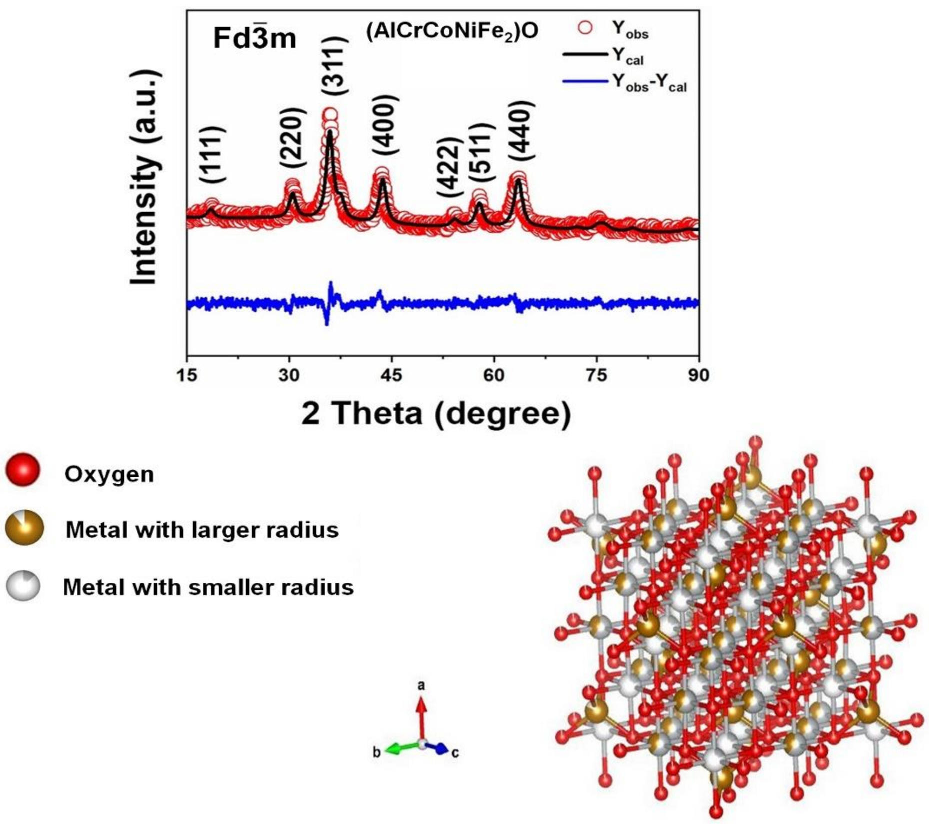

Figure S1. The XRD pattern and the unit cell of ($AlCrCoNiFe_2$)O.

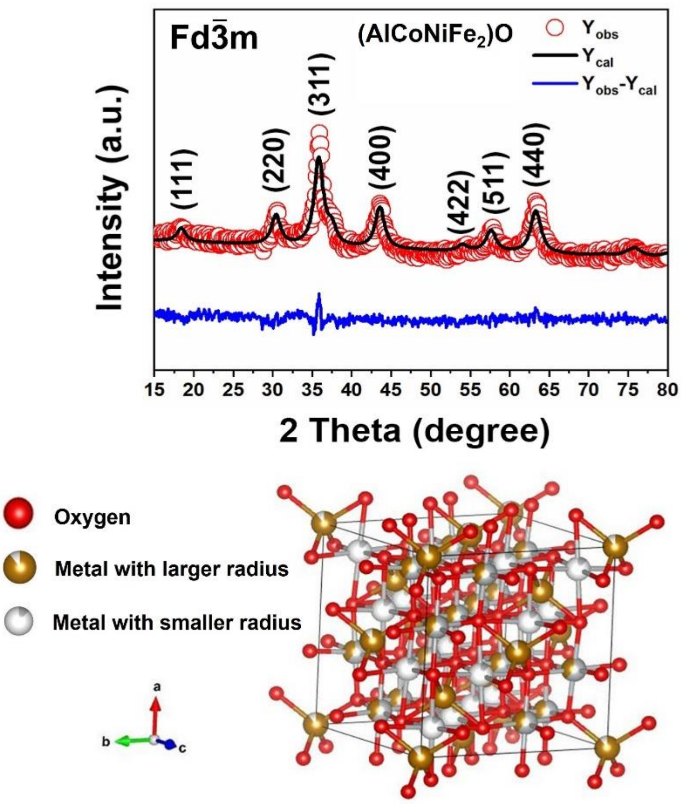

Figure S2. The XRD pattern and the unit cell of ($AlCoNiFe_2$)O.

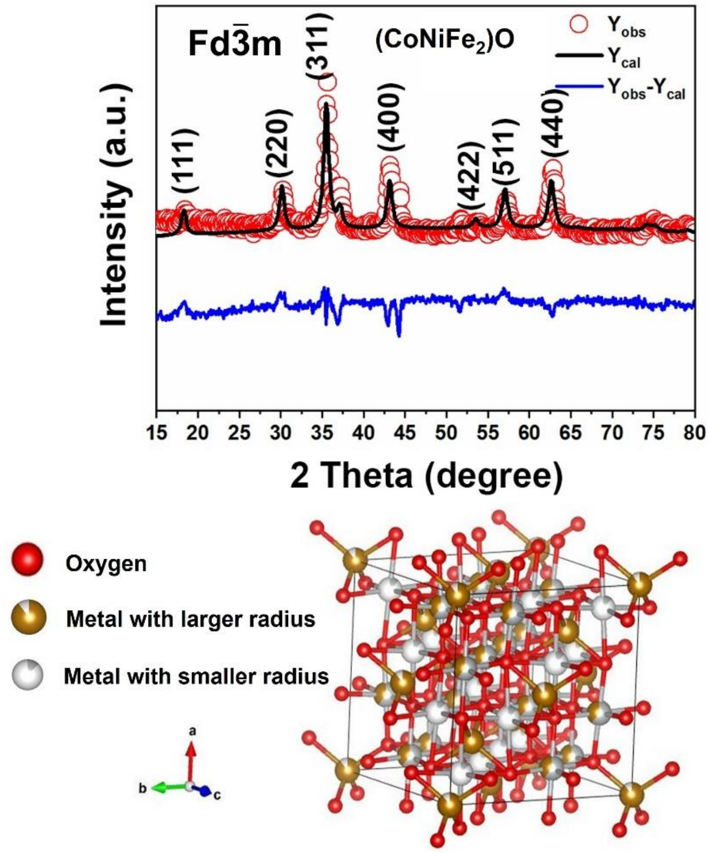

Figure S3. The XRD pattern and the unit cell of **(**CoNiFe$_2$)O.

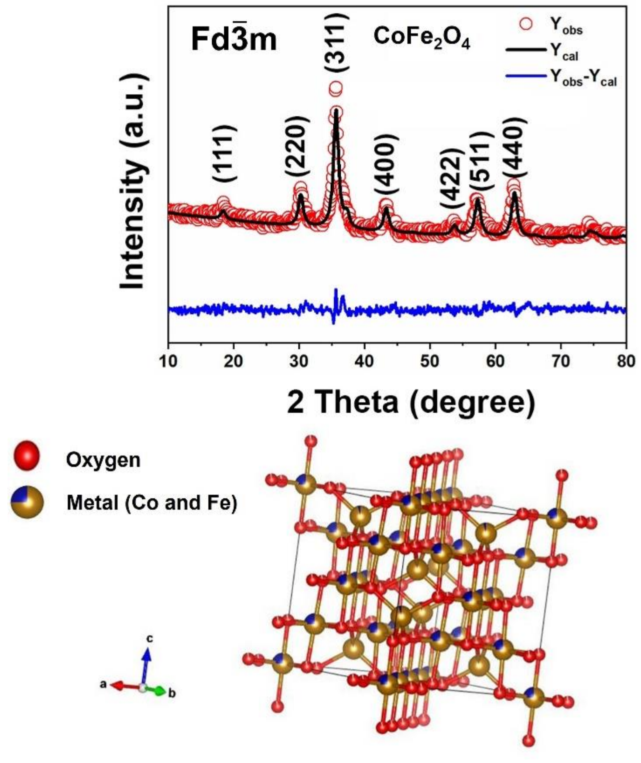

Figure S4. The XRD pattern and the unit cell of CoFe$_2$O$_4$.

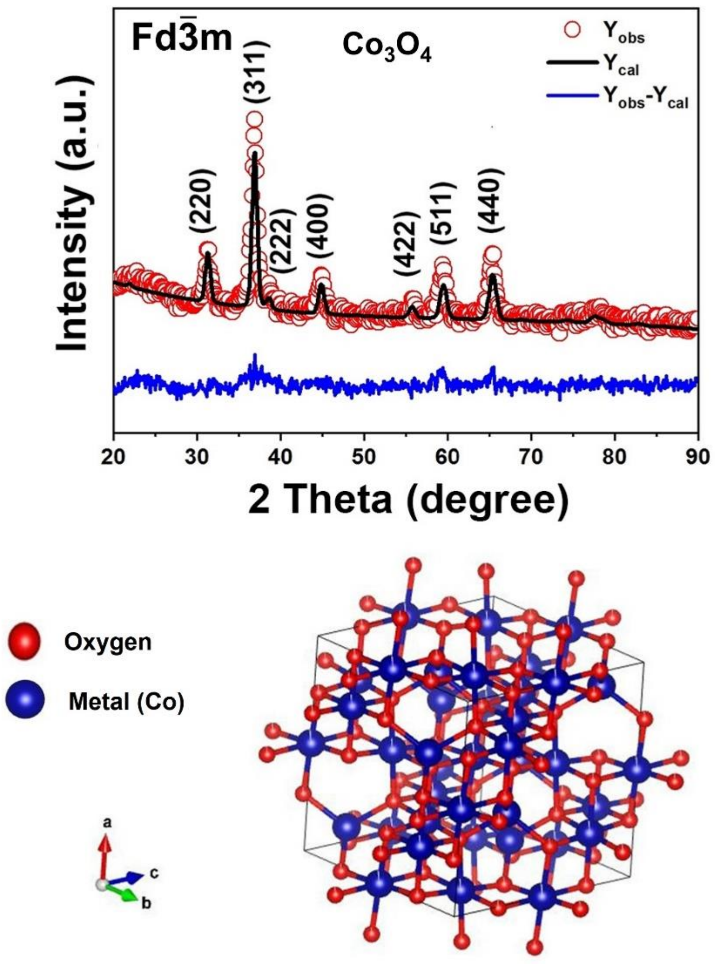

Figure S5. The XRD pattern and the unit cell of Co_3O_4.

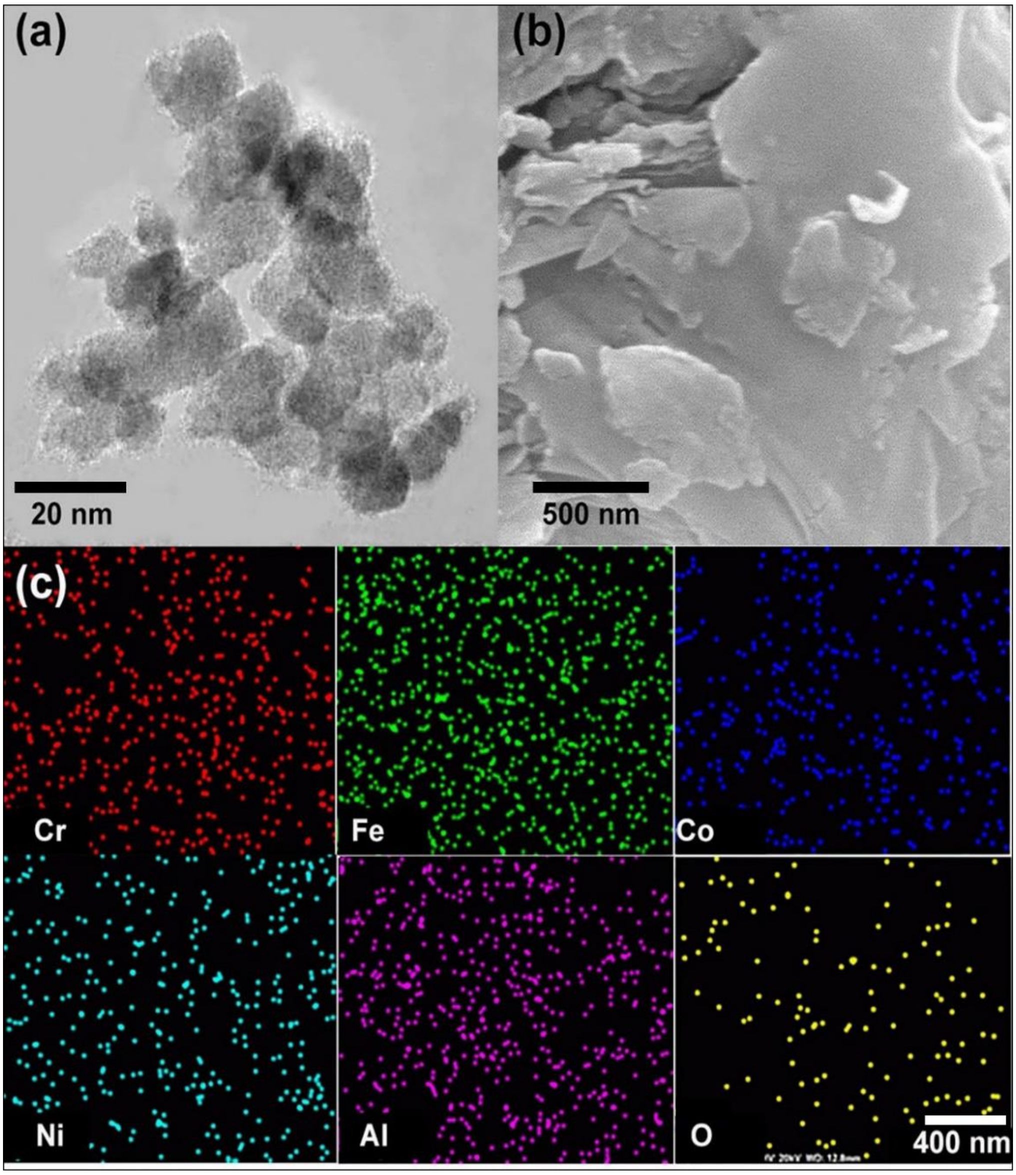

Figure S6. (a) TEM image, (b) SEM and (c) elemental mapping of $(AlCrCoNiFe_2)O$

Table S1. Molar ratios of metals inside multi-metal spinel structures.

**Multi-metal spinel**	**Molar ratio of metals**
$(AlCrCoNiFe_2)O$	(Al:Cr:Co:Ni:Fe)=(1:1:1:1:2)
$(AlCoNiFe_2)O$	(Al:Co:Ni:Fe)=(1:1:1:2)
$(CoNiFe_2)O$	(Co:Ni:Fe)=(1:1:2)
$CoFe_2O_4$	(Co:Fe)=(1:2)

Table S2. Comparison with other works

Electrocatalyst	Electrolyte	Substrate	Mass loading ($mg.cm^{-2}$)	Overpotential (mV)- current density ($mA.cm^{-2}$)	Tafel slope ($mV.dec^{-1}$)	Ref.
**$(AlCrCoNiFe_2)O$**	**1 M KOH**	**Ni foam**	**0.98**	**230-10**	**29**	**This work**
$ZnCo_2O_4$	1 M KOH	Pt	-	390- 10	46	[2]
NiFe-OOH	1 M KOH	Ni foam	Between 0.4 and 50	250-10	36	[3]
NRAHM-NiO	1 M KOH	Ni foam	-	290-100	68	[4]
Ni @Pt core shell nanoplate	1 M KOH	GCE	0.25	290-10	52	[5]
BNHNSs	1 M KOH	CC	-	297-10	100	[6]
LDH-Bir	1 M KOH	-	-	270-10	43	[7]
Fe-Co-2.3Ni-B	1 M KOH	GC	0.3	274-10	38	[8]
NiFe-BTC//G	1 M KOH	-	-	106-10	55	[9]
FNVTiCr	1 M KOH	Ni foam	0.3	240-100	29	[10]
NiCoFe@NiCoFeO NTAs	1 M KOH	CFC	-	201-10	39	[11]
0.27-RuO_2@C	1 M KOH	Carbon matrix	-	240-10	-	[12]
FeNiZn/$FeNi_3$@NiFe-24h	1 M KOH	NiFe foam	-	244-50	45.7	[13]
CS-NiFeCu	1 M KOH	Ni foam	10.2	180-10	33	[14]
NCDC	1 M KOH	Ni foam	2.1	175-50	33	[15]
Ni-Fe-Al	0.1 M NaOH	FTO	-	-	30	[16]
MCPS	0.1 M KOH	-	-	288-10	27.7	[17]
a-LNF (t-d)	1 M KOH	GC	0.232	249-10	36	[18]
Au-MnFeCoNiCu LDH	1 M KOH	CC	1	213-10	27.5	[19]
CoCrNiFeMo HEA	1 M KOH	-	-	220-10	59	[20]
CoFeNiCrMn HEO	1 M KOH	FTO	-	307-10	34.7	[21]
NiFeMo-P	1 M KOH	Ni foam	3.05	196-10	37.8	[22]
HEMP	1 M KOH	GC	-	270-10	52.5	[23]
FeNiCrCoMn-G	1 M KOH	Ni foam	2.5	229-10	40	[24]
$K_{0.8}Na_{0.2}(MgMnFeCoNi)F_3$	1 M KOH	GC	0.14	314-10	55	[25]
Co-$Ni(TCNQ)_2(H_2O)_2$	1 M KOH	GC	0.65	220-10	120	[26]
CoCuFeMoZnAgRu HEO	1 M KOH	Ag foil	-	298-100	30	[27]
CoCuFeMo HEO	1 M KOH	Cu foil	-	199-10	48.8	[28]
FeCoNiCuZn LDH	1 M KOH	CC	-	227-10	41.2	[29]

FeCoNiMg-LDH	1 M KOH	Ni foam	-	302-100	75	[30]
Au-MoS_2/NiO	1 M NaOH	Ni foam	111.1	293-10	100	[31]
Au/NiO	1 M NaOH	Ni foam	111.1	-	74	[31]
β-$Co(OH)_2$	0.1 M KOH	GC	-	-	67	[32]
Dp-$MnCo_2O_4$	1 M KOH	Ni foam	1.3	327-10	79	[33]
Fe-NiHF	1 M KOH	CP	0.3	290-10	44	[34]
hBN	1 M KOH	$NiFeO_xH_y$	-	279-100	30	[35]

[1] Y. Zhang, *Springer Nature Singapore*, **2019**.

http://doi.org/10.1007/978-981-13-8526-1.

[2] J. S. Kim, B. Kim, H. Kim and K. Kang, *Adv. Energy Mater.*, **2018**, *8*, 1702774.

[3] A. Peugeot, C. E. Creissen, D. Karapinar, H. N. Tran, M. Schreiber and M. Fontecave, *Joule*, **2021**, *5*,1281-1300.

[4] K. Hemmati, A. Kumar, A. R. Jadhav, O. Moradlou, A. Z. Moshfegh and H. Lee, *ACS Catal.*, **2023**, *13*, 5516-5528.

[5] F. Wang, G. Chen, X. Liu, F. Chen, H. Wan, L. Ni, N. Zhang, R. Ma and G. Qiu, *ACS Sustain. Chem. Eng.*, **2019**, *7*, 341-349.

[6] J. Mei, J. Shang, T. He, D. Qi, L. Kou, T. Liao, A. Du and Z. Sun, *Adv. Energy Mater.*, **2022**, *12*, 2201141.

[7] X. Long, L. Zhang, Z. Tan and B. Zhou, *Energy Adv.*, **2023**, *2*, 280-292.

[8] J. M. V. Nsanzimana, Y. Peng, Y. Y. Xu, L. Thia, C. Wang, B. Y. Xia and X. Wang, *Adv. Energy Mater.*, **2018**, *8*, 1701475.

[9] S. Lyu, C. Guo, J. Wang, Z. Li, B. Yang, L. Lei, L. Wang, J. Xiao, T. Zhang and Y. Hou, *Nat. Commun.*, **2022**, *13*, 6171.

[10] F. N. I. Sari, G. Frenel, A. C. Lee, Y.-J. Huang, Y.-H. Su and J.-M. Ting, *J. Mater. Chem. A*, **2023**, *11*, 2985.

[11] Y. Liu, Y. Ying, L. Fei, Y. Liu, Q. Hu, G. Zhang, S. Y. Pang, W. Lu, C. L. Mak, X. Luo, L. Zhou, M. Wei and H. Huang, *J. Am. Chem. Soc.*, **2019**, *141*, 8136-8145.

[12] H.-S. Park, J. Yang, M. K. Cho, Y. Lee, S. Cho, S.-D. Yim, B.-S. Kim, J. H. Jang and H.-K. Song, *Nano energy*, **2019**, *55*, 49-58.

[13] Q. Zhou, C. Xu, J. Hou, W. Ma, T. Jian, S. Yan and H. Liu, *Nano-Micro Letters.*, **2023**, *15*, 95.

[14] P. Zhang, L. Li, D. Nordlund, H. Chen, L. Fan, B. Zhang, X. Sheng, Q. Daniel and L. Sun, *Nat. Commun.*, **2018**, *9*, 381.

[15] A. Kumar, S. K. Purkayastha, A. K. Guha, M. R. Das and S. Deka, *ACS Catal.*, **2023**, *13*, 10615-10626.

[16] J. B. Gerken, S. E. Shaner, R. C. Massé, N. J. Porubsky and S. S. Stahl, *Energy Environm. Sci.*, **2014**, *7*, 2376-2382.

[17] Z. Ding, J. Bian, S. Shuang, X. Liu, Y. Hu, C. Sun and Y. Yang, *Adv. Sustainable Syst.*, **2020**, *4*, 1900105.

[18] G. Chen, Y. Zhu, H. M. Chen, Z. Hu, S.-F. Hung, N. Ma, J. Dai, H.-J. Lin, C.-T. Chen, W. Zhou and Z. Shao, *Adv. Mater.*, **2019**, *31*, 1900883.

[19] F. Wang, P. Zou, Y. Zhang, W. Pan, Y. Li, L. Liang, C. Chen, H. Liu and S. Zheng, *Nat. Commun.*, **2023**, *14*, 6019.

[20] J. Tang, J. L. Xu, Z. G. Ye, X. B. Li and J. M. Luo, *J. Mater. Sci. Technol.*, **2021**, *79*, 171-177.

[21] J. Baek, M. D. Hossain, P. Mukherjee, J. Lee, K. T. Winther, J. Leem, Y. Jiang, W. C. Chueh, M. Bajdich and X. Zheng, *Nat. Commun.*, **2023**, *14*, 5936.

[22] X. Zhou, T. Yang, T. Li, Y. Zi, S. Zhang, L. Yang, Y. Liu, J. Yang and J. Tang, *Nano Res. Energy*, **2023**, *2*, e9120086.

[23] D. Lai, Q. Kang, F. Gao and Q. Liu, *J. Mater. Chem. A*, **2021**, *9*, 17913.

[24] T. X. Nguyen, Y.-H. Su, C.-C. Lin, J. Ruan and J.-M. Ting, Adv. Sci., **2021**, *8*, 2002446.

[25] T. Wang, H. Chen, Z. Yang, J. Liang and S. Dai, *J. Am. Chem. Soc.*, **2020**, *142*, 4550-4554.

[26] U. Bhoi, S. Ray, S. Bhand, P. Ninawe, D. Roy, S. Rana, K. Tarafder and N. Ballav, *ACS Energy Lett.*, **2023**, *8*, 4465-4473.

[27] L. Zhang, F. Fan, X. Song, W. Cai, J. Ren, H. Yang and N. Bao, *J. Materiomics*, **2023**. http://doi.org/10.1016/j.jmat.2023.06.006.

[28] L. Zhang, W. Cai and N. Bao, *Adv. Mater.*, **2021**, *33*, 2100745.

[29] J. Yao, F. Wang, W. He, Y. Li, L. Liang, Q. Hao and H. Liu, *Chem. Commun.*, **2023**, *59*, 3719.

[30] D. Liu, X. Yan, P. Guo, Y. Yang, Y. He, J. Liu, J. Liu, J. Chen, H. Pan and R. Wu, *ACS Catal.*, **2023**, *13*, 7698-7706.

[31] R. Ansilda, A. M. C. Devassy, A. Kamalakshan, N. A. Jamuna and S. Mandal, *ASC Appl. Eng. Mater.*, **2023**. http://doi.org/10.1021/acsaenm.3c00534.

[32] M. Kang, C. L. Bentley, J. T. Mefford, W. C. Chueh and P. R. Unwin, *ACS Nano*, **2023**. http://doi.org/10.1021/acsnano.3c06335.

[33] K. Lankauf, K. Cysewska, J. Karczewski, A. Mielewczyk-Gryń, K. Gόrnicka, G. Cempura, M. Chen, P. Jasńki and S. Molin, *Int. J. Hydrogen. Energy*, **2020**, *45*, 14867-14879.

[34] T. Wang, Y. Wu, Y. Han, P. Xu, Y. Pang, X. Feng, H. Yang, W. Ji and T. Cheng, *ACS Appl. Nano Mater.*, **2021**, *4*, 14161-14168.

[35] Y. Lu, B. Li, N. Xu, Z. Zhou, Y. Xiao, Y. Jiang, T. Li, S. Hu, Y. Gong and Y. Cao, *Nat. Commun.*, **2023**, *14*, 6965.
